# Patients with Schizophrenia Do Not Preserve Automatic Grouping When Mentally Re-Grouping Figures: Shedding Light on an Ignored Difficulty

**DOI:** 10.3389/fpsyg.2012.00274

**Published:** 2012-08-17

**Authors:** Anne Giersch, Mitsouko van Assche, Rémi L. Capa, Corinne Marrer, Daniel Gounot

**Affiliations:** ^1^INSERM U666, Department of Psychiatry I, Centre Hospitalier Régional de StrasbourgStrasbourg, France; ^2^Laboratory for Neurology and Imaging of Cognition, University of GenevaGeneva, Switzerland; ^3^Department of Neurology, Geneva University HospitalGeneva, Switzerland; ^4^Laboratoire d’Imagerie et Neurosciences Cognitives, UMR 791 CNRS, Institut de Physique BiologiqueStrasbourg, France

**Keywords:** grouping, visual organization, schizophrenia, top-down grouping

## Abstract

Looking at a pair of objects is easy when automatic grouping mechanisms bind these objects together, but visual exploration can also be more flexible. It is possible to mentally “re-group” two objects that are not only separate but belong to different pairs of objects. “Re-grouping” is in conflict with automatic grouping, since it entails a separation of each item from the set it belongs to. This ability appears to be impaired in patients with schizophrenia. Here we check if this impairment is selective, which would suggest a dissociation between grouping and “re-grouping,” or if it impacts on usual, automatic grouping, which would call for a better understanding of the interactions between automatic grouping and “re-grouping.” Sixteen outpatients with schizophrenia and healthy controls had to identify two identical and contiguous target figures within a display of circles and squares alternating around a fixation point. Eye-tracking was used to check central fixation. The target pair could be located in the same or separate hemifields. Identical figures were grouped by a connector (grouped automatically) or not (to be re-grouped). Attention modulation of automatic grouping was tested by manipulating the proportion of connected and unconnected targets, thus prompting subjects to focalize on either connected or unconnected pairs. Both groups were sensitive to automatic grouping in most conditions, but patients were unusually slowed down for connected targets while focalizing on unconnected pairs. In addition, this unusual effect occurred only when targets were presented within the same hemifield. Patients and controls differed on this asymmetry between within- and across-hemifield presentation, suggesting that patients with schizophrenia do not re-group figures in the same way as controls do. We discuss possible implications on how “re-grouping” ties in with ongoing, automatic perception in healthy volunteers.

## Introduction

We are able to explore and select information in the environment in a flexible way and usually do not experience any limits or difficulty when doing so. In a cluttered visual scene, we can mentally select and extract visual information and even relate objects that have nothing in common. This ability appears to be impaired in patients with schizophrenia, and may impact on how they adapt to the visual environment. It has been related to a more general difficulty at organizing information that is expressed at a clinical level (Silverstein and Keane, 2011). However, the mechanisms of these impairments are still debated, and especially the relative contribution of automatic grouping mechanisms vs. high-level, top-down mechanisms. Our aim is twofold. Understanding how patients with schizophrenia explore the visual environment should help us to understand the mechanisms underlying their difficulties when attempting to adapt to an ever changing environment. More generally it might contribute to objectify and better define the difficulties of patients at organizing information. Second, patients’ results lead to questions regarding the mechanisms of the mental selection of objects in healthy volunteers and how these mechanisms tie in with automatic grouping. This question is not fully resolved in healthy controls. For this reason we went back and forth from fundamental knowledge to clinically related issues. We explored the ability to mentally relate objects in healthy volunteers, and we use this same paradigm here in patients with schizophrenia. The results will be used to discuss first their significance for patients and second what they reveal about mental selection and visual organization in healthy volunteers.

There is already considerable knowledge regarding visual processing in healthy volunteers. Form processing is known to involve a number of steps, from the extraction of primitives (local orientation, color, luminance, etc.), to the integration of the form contour and surface filling-in that sub-tend object recognition (Boucart et al., 1994; Humphreys, 2003; Grossberg et al., 2007). The integration of contour information involves Gestalt rules like grouping by collinearity, proximity, similarity or common fate, and the use of segmentation cues in order to correctly separate object parts, objects from the background, and objects from one another (Boucart et al., 1994; Kovács, 1996; Beck and Palmer, 2002; Spillmann, 2006). Similar rules apply when considering the relationship between distinct objects, even though the pathways sub-tending the coding of relations between objects are distinct from those sub-tending the coding of the relations within objects (Humphreys, 1998; Davis, 2001). Grouping between individual items allows to identify global forms that emerge from the way local elements are organized (Kimchi, 2000; Kimchi et al., 2005). Information at the global and local levels are processed by specialized neural pathways, and structure the visual environment in a hierarchical manner (Delis et al., 1986; van Kleeck, 1989; Hübner and Volberg, 2005). A number of studies suggest that grouping mechanisms can occur automatically under conditions of inattention (Driver et al., 2001; Müller et al., 2010). It has been shown also, however, that attention can interact with grouping (Driver et al., 2001), and can be directed either toward the local or the global level (Robertson et al., 1993; Humphreys, 1998).

**Figure 1:**
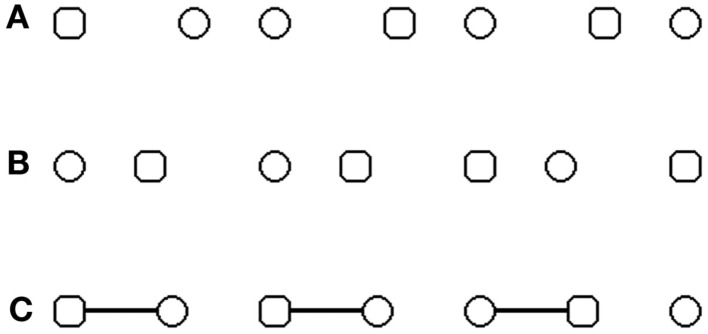
**Illustration of the stimuli used to explore visual grouping and re-grouping**. **(A–C)** stimuli used in the original paradigm of Beck and Palmer (2002), with a manipulation of grouping by proximity **(A,B)** and by connecters **(C)**. Only two shapes shared the same form and were displayed one beside the other. These two shapes represent the targets. Subjects had to decide if the two targets are two circles or two squares. **(A)** Example of circle targets belonging to the same pair of figures. **(B)** Example of square targets belonging to different pairs of figures. **(C)** Example of circles targets belonging to different pairs of figures.

Here, we question what happens when attention is directed toward object pairs that are unrelated and do not form a global shape. We argue this question is not resolved by usual mechanisms of grouping, and we suspect it might be crucial to understand the impairments in patients with schizophrenia (van Assche and Giersch, 2011). We explore it by using a paradigm designed by Beck and Palmer (2002). Beck and Palmer (2002), see also Palmer and Beck (2007) built visual search tasks with a setting which can be considered as a simplified version of a visual scene. Squares and circles represent simple objects and are displayed on a horizontal row. Squares and circles alternate on the row, except for two shapes sharing the same form and displayed one beside the other (Figures 1A–C). The task of the subjects is to spot these two identical and adjacent shapes, which represent the targets, and to discriminate their form, i.e., to decide whether they are two circles or two squares. An additional manipulation allows us to evaluate the effect of grouping. The objects on the row are grouped by pairs on the basis of classical rules like proximity, or the presence of connectors linking the shapes. As a consequence of this grouping manipulation, the two identical shapes are either part of the same pair of related figures (i.e., grouped by proximity or connecters), or part of different pairs (i.e., unrelated). As can be expected, it is easier for subjects to find the targets if they are part of a pair of related shapes (i.e., grouped), than if they are unrelated and part of different pairs. This effect reflects the advantage provided by grouping. Interestingly, this advantage is modulated by contextual information, i.e., the percentage of related vs. unrelated targets within an experimental block. Beck and Palmer (2002) used three experimental blocks, one with 75% unrelated and 25% related targets, one with equal proportions of unrelated and related targets, and one with 25% unrelated and 75% related targets. The advantage for related targets increases when related targets are the majority, and decreases when they are the minority. This modulation could not be explained by repetition effects, i.e., facilitated search for a target pair when it follows a trial with a pair in the same condition (e.g., related targets following related targets or unrelated targets following unrelated targets). Indeed, Beck and Palmer (2002) observed probability effects for both repeated and non-repeated trials. The performance modulation rather reflects the prioritization of one type of pairs (related or unrelated) according to the contextual information provided by the frequency of these pairs within a given experimental block. On each trial subjects must process visual information in order to locate the related pairs, and then can direct their attention to the prioritized pairs. Since the prioritization relies on the estimation of a frequency across different trials and is not provided by information in a single trial, it can thus be considered as a top-down effect. This does not mean that subjects provide a conscious effort to prioritize related or unrelated pairs. When subjects are not informed about the proportion modulation and cannot report it after the tasks, the effect is nonetheless identical to the prioritization obtained when subjects are informed (data obtained in unpublished pilot studies). All in all the modulation is considered top-down because it results from a global and automatic probability estimation rather than from a local priming effect.

A top-down modulation of grouping does not imply that unrelated targets can be prioritized, and as a matter of fact, the possibility to prioritize unrelated targets is not straightforward. In the results of Beck and Palmer (2002), the modulation effects for related and unrelated shapes were usually symmetrical. This means that each time performance was improved for related pairs, there was a symmetrical cost for unrelated pairs, and the reverse. Such results can be interpreted as a modulation of the prioritization of connected pairs, and the performance variations for unrelated shapes might be an automatic consequence of the varying prioritization of connected pairs. The more subjects would focus on connected pairs the less they would spend on unrelated shapes. In other words, the results do not imply that unrelated shapes are prioritized selectively. As a matter of fact, the advantage for related over unrelated shapes shows that selecting two unrelated shapes at the same time entails some difficulties (Beck and Palmer, 2002). The literature on multiple object tracking confirms these difficulties, even though it shows it is possible to select distinct shapes. During multiple object tracking tasks, subjects select several unrelated objects efficiently enough to track them when they move in distinct directions among distracters (reviews in Pylyshyn, 2001; Cavanagh and Alvarez, 2005; Alvarez, 2011). It has been proposed that such ability is sub-tended by goal-directed re-grouping of the separate objects (Yantis, 1992; Alvarez, 2011). However, this ability is severely impacted when the selected objects are automatically grouped with distracters (Scholl et al., 2001; Suganuma and Yokosawa, 2006). This suggests that it is very difficult to select distinct objects when each one is part of a different group. Even with a simpler visual search paradigm and static objects, object-centered attention can be expected to induce difficulties when trying to focus on two shapes that belong to different pairs of objects. Object-centered attention implies that when attention is focused on an element of a group, then attention spreads to the whole group (Duncan, 1984; Egly et al., 1994; Matsukura and Vecera, 2006). This means that when distracters are grouped with target information, attention directed toward the target will spread to distracter information, and attention is not drawn on target information in a selective way anymore. Despite this, is it really the case that we cannot attend selectively to two items when they belong to different sets of objects? In every day life, it can happen that we pick up detail information in different sets of objects and compare them or associate them mentally. In fact, it happens each time information is hierarchical, and we wish to associate mentally details from different hierarchical objects sets (e.g., flowers from different houses, leaves from different trees, fruits from different piles). Yet we are usually able to compare two details from different houses, fruit piles, or trees without experiencing any noticeable difficulty. Our own results (Giersch and Rhein, 2008; van Assche et al., 2012) confirm we can attend to such details and associate them selectively.

**Figure 2:**
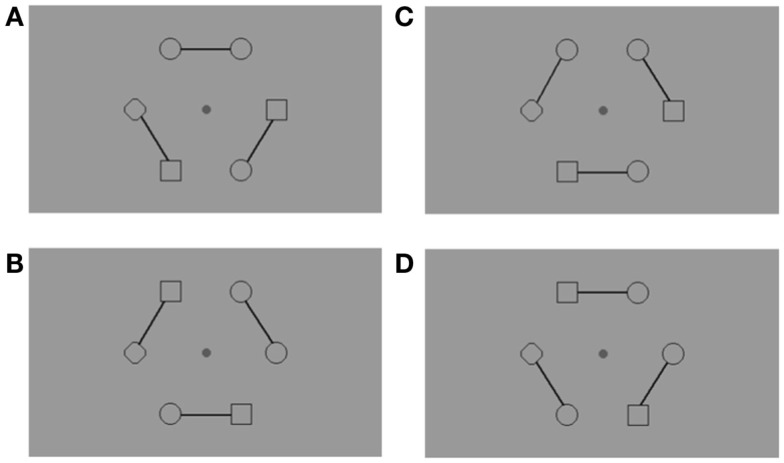
**Example of the stimuli used with an arrangement of figures around a central fixation point**. Subjects had to fixate the central point throughout the trials, and this was checked with continuous eye-tracking (Cambridge Research System, 50 Hz). Connecters were introduced to link elements in pairs. Consequently, the target pair could be either connected across-hemifields **(A)**, connected within the same hemifield **(B)**, unconnected across-hemifields **(C)**, or unconnected within the same hemifield **(D)**. There was always a diamond (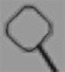) on the horizontal meridian, which remained in the same location during a block of trials, either in the right or the left hemifield. Subjects were informed about the position of the diamond before the experimental block. The diamond ensured that (1) only two adjacent figures were identical, and (2) the target pair was located in equal proportion in the across-hemifield and within-hemifield conditions.

We will call “re-grouping” the ability to attend selectively to figures that are not only separate but also part of different sets of objects. We obtained some evidence of “re-grouping” by deriving new paradigms from the one elaborated by Beck and Palmer (2002). We observed in two different studies that healthy volunteers are able to focus selectively on unrelated pairs, even when they belong to different pairs of figures. (Giersch and Rhein, 2008; van Assche et al., 2012). In van Assche et al. (2012), targets were circles and squares like in the original paradigm, but they were arranged in alternation on a circle around a fixation point. Subjects decided whether the two identical shapes located one beside the other were two circles or two squares, as in the typical experiment. The presence of connecters led to the perception of pairs of figures (Figure 2), and as in the previous experiments, targets were either part of the same connected pair, or belonged to two different pairs. We manipulated the frequency of connected and unconnected targets in three different experimental blocks. Contrary to previous experiments however, subjects were instructed to look at the central fixation point throughout the experiment, and this was checked by continuous eye-tracking. In case of an ocular saccade out of central area, the trial was stopped, and was presented again at the end of the experimental block. Hence, subjects could not visually sweep across the stimuli. Because eye movements were not allowed, subjects could not compare nearby figures through ocular exploration, and had to relate them mentally. This might explain why this procedure helped us to evidence “re-grouping” of unrelated figures more easily than previous paradigms. As a matter of fact, the results showed that subjects became significantly faster (by no less than 123 ms) at finding unconnected targets when those targets were the majority, as compared to the block with an equal proportion of connected and unconnected targets. Despite this large improvement, performance for connected targets remained stable across these two blocks, suggesting that focalization on unconnected targets cannot be explained by an inhibition of connected targets and rather involve a selective “re-grouping” of unconnected targets.

All in all, the results suggest that unrelated stimuli can be isolated and “re-grouped” efficiently, even if they belong to different objects groups. This mechanism bypasses object-centered attention and cannot be accounted for by global/local processing. Local and global information correspond to individual shapes and pairs of related figures, respectively, but the pairs of unrelated figures correspond to neither, and may require higher-level cognitive operations. A late mechanism would be dissociated from usual mechanisms of visual grouping and would rather involve attentional selection mechanisms. It should be noted that our data does not allow us to distinguish between a simultaneous selection of two stimuli and the possibility that each figure is attended to in turn very fast, i.e., that items are selected sequentially rather than simultaneously (Hogendoorn et al., 2010). In the latter case (sequential selection), subjects would not be conscious of alternating between items. Hence, this possibility is beyond the scope of this paper, since both possibilities allow for a selective focalization on the two figures during a period of time. As such, both possibilities, simultaneous selection or fast serial selection, lead to questions regarding object-based attention, and conflict with usual, automatic grouping. We explored this question further by studying to which extent the outputs of automatic grouping and “re-grouping” differ.

Even if the mechanisms underlying this ability are clearly different to the ones underlying classical grouping, one might wonder whether they have comparable end-products.

We used the cost of across-hemifield presentation as a tool to contrast the impact of automatic grouping and “re-grouping.”

We observed that connecters between targets, or physical arrangement leading to automatic grouping, erased the cost of across-hemifield presentation (van Assche et al., 2012). The benefit provided by connecters is akin to what had been described in patients with parietal lesions, who display a difficulty to perceive stimuli in the contra-lesional hemifield (Driver, 1995; Gilchrist et al., 1996; Pavlovskaya et al., 1997; Boutsen and Humphreys, 2000; Brooks et al., 2005). The benefit of grouping contrasted with “re-grouping,” which was without effect on this cost. Even when attention prioritization led to a large improvement of performance for unconnected targets, the cost of across-hemifield presentation remained high. In fact, it was as high as when unconnected targets were the minority. These results suggest that in addition to taking different routes, grouping and “re-grouping” also differ in their output. In other words, outputs for “re-grouping” and automatic grouping would differ. This would sub-tend our subjective experience suggesting that automatic grouping provides background information and that our mental exploration is akin to playing with such information at the foreground. However, as emphasized above, we perceive only one unique outer world, implying that “re-grouping” must be somehow tied in with automatic grouping.

The literature and our own results suggest that understanding the role of “re-grouping” and how it ties in with automatic grouping might be crucial in patients with schizophrenia. Conversely, the results in patients might shed light on this question. A number of studies has shown that patients with schizophrenia have a difficulty to organize visual information (review in Silverstein and Keane, 2011), and our own initial studies suggested a selective difficulty to “re-group” unconnected items (Giersch and Rhein, 2008; van Assche and Giersch, 2011). A selective difficulty at “re-grouping” would be an argument in favor of a complete dissociation between automatic grouping and “re-grouping.” Recently however, we used a working memory task, and results suggested that patients can re-group items when incited to, but then experience a conflict between usual grouping and “re-grouping,” which contrasts with results in controls. This suggests that the difficulty at “re-grouping” also impacts on the ability of the patients to use automatic grouping processes. If grouping and “re-grouping” are found to be competing in patients but not in controls, this would confirm that the usual preservation of automatic grouping is not as straightforward as believed. It would call for explanations on how healthy subjects avoid this competition and make “grouping” and “re-grouping” coexist.

The results to date were obtained in a memory task, however, and the competition between representations of related and unrelated figures might have been specific to this memory task. To test this possibility, we checked whether similar results could be obtained in a perception task.

In order to test the possibility of a competition between grouping and re-grouping in patients with schizophrenia, we used again figures arranged in a circle around a central fixation point, as already described. We chose this arrangement because it had been particularly efficient in showing the effect of a prioritization of unrelated figures in healthy subjects (van Assche et al., 2012). If patients are unable to re-group items, then we should see no effect of prioritization in patients, i.e., less variation in performance than in controls when the proportion of related and unrelated figures is manipulated. These results would then be similar to those observed in our first study (Giersch and Rhein, 2008). If on the contrary the task is efficient in inciting patients to re-group unrelated figures, then we should observe performance variations across blocks. Most importantly, if patients can maintain the link between related figures while re-grouping information, then performance for related figures should be preserved. If in contrast patients can re-group unrelated figures only at the expense of the link between related figures, as we observed recently (Giersch et al., 2011) then we should observe a cost for related figures that is symmetrical to the gain for unrelated figures. This would indicate a competition between the access to related and unrelated figures in patients, and would reinforce our hypothesis that specific mechanisms are at work to enable the coexistence of the two types of groupings.

In addition, we contrasted within- and across-hemifield presentations, and this was expected to further our understanding of the mechanisms at work, and especially to what extent automatic grouping and “re-grouping” are dissociated. Our previous work has shown that our configuration leads to a large RT cost in case of unconnected targets displayed across-hemifields. Interhemispheric transfer is believed to be impaired in patients (Schwartz et al., 1984; Mohr et al., 2008, but see David, 1993), and we wondered if this explains the difficulties of patients with schizophrenia at “re-grouping.” In that case patients with schizophrenia should be impaired relative to controls mainly in case of across-hemifield presentation. On the other hand, if patients can focus on re-grouped figures, the comparison of the effects of hemifield presentation in patients and controls was expected to give some indications on the mechanisms at work in the two groups. The idea was that early mechanisms of “re-grouping” were expected to be sensitive to the cost of interhemispheric transfer, whereas later and lateralized mechanisms (Kosslyn, 1987; van der Ham et al., 2011; Stevens et al., 2012) would be less sensitive to this cost. Our previous results confirmed that even though the presentation across-hemifields globally slowed down healthy subjects when the targets were unconnected, it was without effect on the prioritization induced by the manipulation of the percentage of connected vs. unconnected figures. If patients with schizophrenia re-group and prioritize pairs the same way as controls, then their pattern of responses should be similar. In contrast, a difference in the effect of across-hemifield presentation might reveal a difference in the mechanisms at work in the two groups.

## Materials and Methods

### Participants

Sixteen outpatients responding to the DSM IV criteria for schizophrenia took part in this study. The diagnosis was based on a semi-structured interview (the *Mini International Neuropsychiatric Interview*) and established by a senior psychiatrist of the University Psychiatry Department. Symptoms were assessed by means of the Positive and Negative Syndrome Scale (PANSS, Kay et al., 1987). Patients were matched with 16 healthy subjects on age, sex, and education level (Table 1). One control subject was discarded from analysis, due to technical problems with the response recording and thus 15 healthy subjects remained.

**Table 1:** **Demographic and clinical data of the participants**.

	Patients (*N* = 16) mean ± SD	Controls (*N* = 15) mean ± SD	Group comparison
Gender (M/F)	12/4	11/4	
Age	31.8 ± 6	31.3 ± 6.3	*t*(29) < 1, ns
Years of education	12.5 ± 2.6	12.8 ± 1.9	*t*(29) < 1, ns
Age at onset	23.5 ± 4.8		
Disease duration	8.5 ± 5.5		
Mean number of hospitalizations	1.7 ± 1.7		
Medication (typical/atypical/+antiparkinsonian/no medication)	3/11/1/1		
Dose of medication in chlorpromazine equivalent	259 ± 164		
PANSS total	76.2 ± 21		
PANSS positive sub-scale	17.4 ± 4.9		
PANSS negative sub-scale	20.3 ± 6.4		
PANSS general sub-scale	38.4 ± 12.2		

Subjects had normal or corrected-to-normal visual acuity, and were right-handed according to the Edinburgh inventory (Oldfield, 1971). They had no history of neurological disorder, generalized anesthesia within the past 3 months, drug abuse or benzodiazepines medication. All participants gave written informed consent prior the beginning of the study, consistently with the recommendations of the Declaration of Helsinki. This project was approved by the local ethics committee.

### Stimuli

Each display contained six figures (0.5° × 0.5° of visual angle each). Circles and squares were positioned along a virtual circle (diameter = 6.8°) centered on a central fixation point. Circles and squares were displayed in spatial alternation except for two figures, the target pair, which were identical, and a single diamond. Unlike the circles and squares, the diamond was always in the same location on the horizontal meridian during a block of trials, either in the right or the left hemifield. Subjects were informed about the position of the diamond before the experimental block. This display configuration, and especially the diamond, ensured that (1) only two adjacent figures were identical, and (2) the target pair was located in equal proportion in the across-hemifield and within-hemifield conditions. There were two possible target locations for the across-hemifield location, and two possible target locations for the within-hemifield location, one above and one below the fixation point.

Three solid connectors linked figures by pairs (Figure 2). The targets could thus be within the same perceptual group (connected targets) or between two perceptual groups (unconnected targets). In each connected and unconnected condition, targets were displayed equally often in the same hemifield or across different hemifields.

### Procedure

Subjects were instructed that they had to look for two target shapes that were identical and displayed one beside the other. Their task was to identify whether the two targets were two circles or two squares and to answer by pressing on a right (two circles) or left (two squares) response key, respectively. The onset of the display activated the computer clock, which was stopped when the subject pressed a key. Subjects were shown several examples on paper to illustrate the different target locations, and to ensure that they did not ignore unrelated targets. The distinction between related and unrelated targets was not made explicit, however.

Subjects were further told to continuously gaze at the central fixation point throughout the experiment. This ensured that our targets were effectively displayed in the same or different hemifields, and thus processed in the same or in different brain hemispheres, respectively. In addition, central fixation impeded subjects from visually sweeping across the stimulus, forcing them to covertly attend to the figures pairs instead (Moore et al., 2003; Herrington and Assad, 2010). This represents a major difference relative to previous studies with patients with schizophrenia (Giersch and Rhein, 2008; van Assche and Giersch, 2011), but is similar to our previous study in healthy volunteers (van Assche et al., 2012).

### Eye-tracker

Eye position was recorded throughout the experiment to check constant fixation of the central point (ASL monocular infrared eye-tracker; sampling rate: 50 Hz).

### Experimental design

The experiment was part of a protocol in fMRI; here we focus on two experimental blocks realized inside the scanner and designed to bias subjects toward connected or unconnected targets. For the sake of simplicity we do not present the results of the experimental blocks used to measure brain activation as a function of the target type. In each of the two blocks analyzed here, the proportion of the target types was manipulated. One block biased subjects toward unconnected targets (75% unconnected +25% connected targets) whereas the other block biased subjects toward connected targets (75% connected +25% unconnected targets).

All subjects were first trained extensively outside the scanner to ensure that they would be able to fixate the central fixation point throughout the scanner session, and thus that they would search for the targets covertly and not overtly. Our main aim was to examine the impact of the bias toward unconnected targets, and all subjects started with the experimental block with a majority of unconnected targets. The sequence of the two experimental blocks was repeated twice in the same order. The first and second run were identical, except that the location of the diamond differed (in the right vs. left hemifield, the order of the two runs being randomized across participants). Subjects were not told about the manipulation of the proportion of each target type. We checked the impact of the instructions in a preliminary experiment in healthy volunteers, and showed that performance was identical when subjects were told or not about the proportion manipulation. We preferred not to give information about the manipulation in order to avoid a possible difference in the use of this knowledge between groups. As emphasized above, it should be noted that subjects are unable to report the manipulation when asked at the end of the experiment, suggesting that the prioritization does not require a conscious effort. After the fMRI setup up, instructions were displayed on the screen, followed by an eye-tracking calibration. The validity of this calibration was checked before the beginning of the second run. Each trial began with the presentation of a central fixation point for 500 ms. The six figures appeared around this fixation point for 5000 ms, with only two adjacent figures being identical and representing the targets. Inter-trial duration was 500 ms. There were a total of 224 trials. Here we will report only the behavioral data.

### Behavioral data analysis

Median RTs were derived from individual performance. ANOVAs were conducted on RTs and error rates. Trials with error were removed from RTs analysis.

Within-subject factors were the target type (connected vs. unconnected), hemifield presentation (across- vs. within-hemifield) and target type proportion (block with a bias toward unconnected vs. toward connected targets). The group (patients vs. controls) was the between-subjects factor. Data were pooled across the two runs (all interactions between runs – 1st vs. 2nd – and other factors – target type, hemifield presentation, target type proportion and groups: *F*s < 1; there was no effect of right vs. left presentation).

## Results

**Figure 3:**
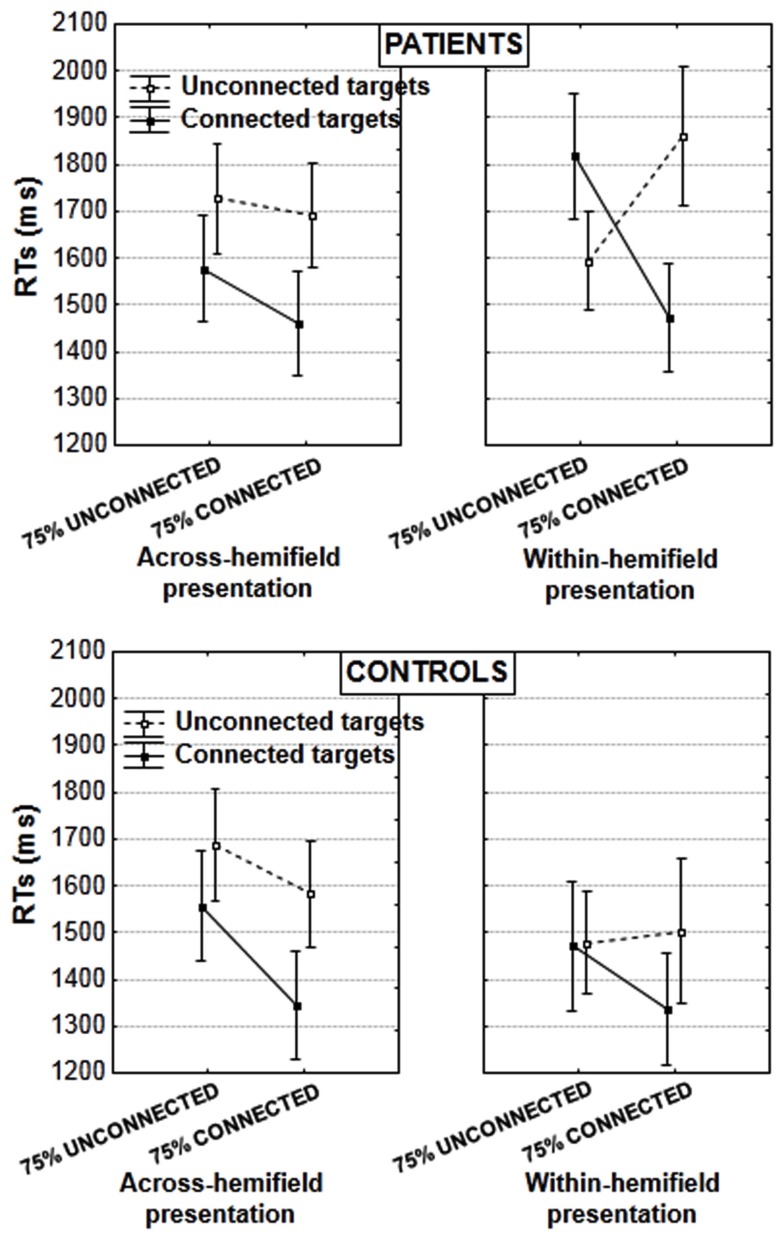
**Mean RTs in the induction blocks, in patients (upper panel) and controls (lower panel; vertical bars: error bars)**. Results are illustrated according to the experimental blocks (with a bias toward unconnected vs. connected figures), type of target pair (unconnected vs. connected), and presentation of target pair (in the same or in different hemifields).

**Figure 4:**
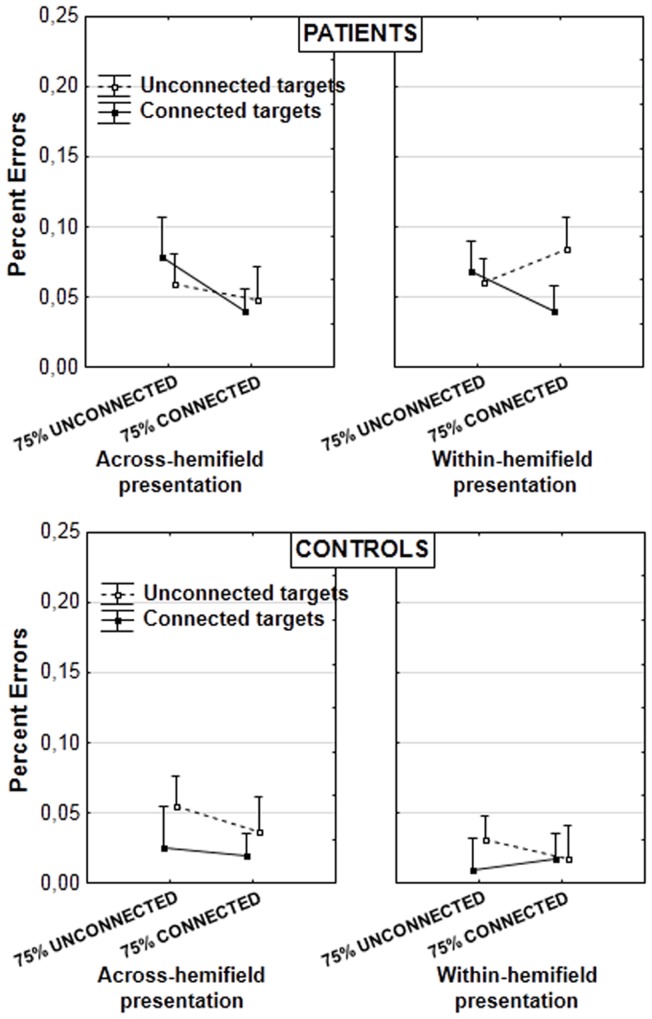
**Mean percent errors in patients (upper panel) and controls (lower panel; vertical bars: error bars)**. Results are illustrated according to the experimental blocks (with a bias toward unconnected vs. connected figures), type of target pair (unconnected vs. connected), and presentation of target pair (in the same or in different hemifields).

There was no main effect of group: patients were only slightly slower and less accurate than controls [by 155 ms, *F*(1, 29) = 1.030, *p* = 0.318, partial η^2^ = 0.034, and 3.3%, *F*(1, 29) = 2.364, *p* = 0.135, partial η^2^ = 0.075]. There was however a significant group × target type × target type proportion × hemifield presentation interaction on RTs [*F*(1, 29) = 4.459, *p* = 0.043, partial η^2^ = 0.133], and a target type × target type proportion × group interaction on percent errors [*F*(1, 29) = 8.555, *p* = 0.006, partial η^2^ = 0.228]. We first detail RTs (illustrated in Figure 3). We then estimate the cost of across-hemifield presentation and summarize data on error rates (Figure 4).

Patients and controls differed when targets were in the same hemifield [target type × target type proportion × group interaction: *F*(1, 29) = 6.353, *p* = 0.017, partial η^2^ = 0.179]. The interaction between target type and target type proportion was significant in patients [*F*(1, 15) = 17.160, *p* = 0.0009, partial η^2^ = 0.533] but not in controls [*F*(1, 14) = 2.518, *p* = 0.135, partial η^2^ = 0.152]. Patients showed an unusual advantage for unconnected relative to connected targets in the block with a bias toward unconnected figures [by 224 ms, *F*(1, 15) = 5.376, *p* = 0.035, partial η^2^ = 0.264, Figure 3 rightward upper panel]. This was not the case in controls, who were equally fast for connected and unconnected targets in the block with a bias toward unconnected figures [*F*(1, 14) = 0.009, *p* = 0.926, partial η^2^ = 0.0006]. In contrast in the block with a bias toward connected targets, the advantage for connected over unconnected targets was significant in both patients [390 ms, *F*(1, 15) = 10.621, *p* = 0.005, partial η^2^ = 0.414] and controls [165 ms, *F*(1, 14) = 6.649, *p* = 0.022, partial η^2^ = 0.322; these effects do not differ significantly, *F*(1, 29) = 1.525, *p* = 0.227, partial η^2^ = 0.050]. To summarize, patients showed a significant advantage for unconnected over connected targets when the former were the majority, and vice versa. On the other hand, controls performed equally for both target types when unconnected targets were the majority, showing that despite the priority toward unconnected targets, they were still efficient in occasional trials with connected targets.

When targets were displayed in different hemifields, there was no interaction with group [target type × target type proportion × group interaction: *F*(1, 29) = 0.032, *p* = 0.859, partial η^2^ = 0.001]. Both patients and controls showed the typical advantage for connected over unconnected targets (controls: 184 ms [*F*(1, 14) = 22.168, *p* = 0.0003, partial η^2^ = 0.613], patients: 190 ms [*F*(1, 15) = 12.581, *p* = 0.003, partial η^2^ = 0.456]. This replicates results obtained with another sample of healthy subjects, and shows that connectors linking targets across-hemifields allows to facilitate the comparison of targets that are initially processed in different brain hemispheres (van Assche et al., 2012).

As a result of the performance differences in case of within- and across-hemifield presentation in patients, there was a significant interaction between target type (connected vs. unconnected), hemifield presentation (across- vs. within-hemifield), and target type proportion (with a bias toward unconnected vs. toward connected targets) in the patients’ group: *F*(1, 15) = 11.752, *p* = 0.004, partial η^2^ = 0.439. In controls, there was a global effect of hemifield presentation [with an advantage of 96 ms for within- vs. across-hemifield presentation, *F*(1, 14) = 5.453, *p* = 0.035, partial η^2^ = 0.280] but no interaction with other effects: especially, there was no interaction between target type, target type proportion, and hemifield presentation [*F*(1, 14) = 0.119, *p* = 0.734 partial η^2^ = 0.008].

It should be noted also that the difficulties displayed by patients with schizophrenia in case of a within-hemifield presentation make it difficult to estimate the cost of across-hemifield presentation. Patients with schizophrenia were rather faster in case of across-rather than within-hemifield presentation, by 72 ms, when all results are averaged. This effect was not significant [*F*(1, 15) = 1.409, *p* = 0.254, partial η^2^ = 0.086] but differed significantly from the opposite effect observed in controls [*F*(1, 29) = 5.097, *p* = 0.032, partial η^2^ = 0.149]. To evaluate the cost of across-hemifield presentation more closely, we additionally checked the effect of hemifield presentation for the two conditions yielding the fastest responses in patients when the targets were in the same hemifield. For connected targets in the block with a bias toward connected pairs, there was no significant effect in either group, and no interaction between group and hemifield presentation [*F*(1, 29) = 0.110, *p* = 0.732 partial η^2^ = 0.004]. For unconnected targets, there was a cost of across-hemifield presentation in both groups (209 ms in controls and 133 ms in patients with schizophrenia) and this cost did not differ across groups [*F*(1, 29) = 0.489, *p* = 0.490 partial η^2^ = 0.017]. No effect involving the hemifield presentation was found significant in the analysis on error rates.

In the analysis on error rates, we decomposed the already mentioned interaction target type × target type proportion × group [*F*(1, 29) = 8.555, *p* = 0.006, partial η^2^ = 0.228]. A significant interaction between target type and target type proportion was found in patients [*F*(1, 15) = 6.212, *p* = 0.025, partial η^2^ = 0.293]. Further decompositions did not yield significant effects however. Nor was there any significant effect in controls. The effect could thus be attributed to opposite trends of grouping across experimental blocks in patients (Figure 4). The graph suggests it is present mainly when targets are displayed within the same hemifield, but this is not supported by statistical analyses. The lack of randomization across blocks and the lack of significant effects after decomposition of the results mean these results should be taken with caution. They do not contradict the results on RTs, however.

## Discussion

The results show that patients can re-group two items which belong to different perceptual groups when incited to do so. Patients improve performance for unconnected targets when those are more frequent, unlike in our previous studies (Giersch and Rhein, 2008; van Assche and Giersch, 2011), suggesting that they can re-group items under specific task conditions. However, this is accompanied by a disadvantage for connected targets in the within-hemifield condition, replicating results with a memory-related paradigm (Giersch et al., 2011). Being slower for connected than unconnected targets is unusual. We observed this effect once in controls, but only in untrained subjects (van Assche et al., 2012), which was not the case here. All subjects were trained extensively before the test, and in healthy subjects, the results always show a preserved access to connected figures, even when the task incites subjects to prioritize unconnected figures efficiently.

It is as if controls have access to connected targets whatever the attention conditions. In contrast, re-grouping unconnected targets leads patients to temporarily lose the perceptual organization derived from automatic grouping. Several limitations should be discussed first, however.

There is one methodological limitation to this work related to the fact that all subjects started with a block driving them to prioritize unconnected figures. This was done for imaging reasons and prevented us from directly comparing performance variations across blocks. It is to be noted, however, that the opposite effects for connected and unconnected targets across blocks (in case of within-hemifield presentation in patients) cannot be explained by effects of order: for connected targets, performance improves from one block to the other, whereas the opposite is observed for unconnected targets. Most importantly, we base our analysis on performance differences observed within a single experimental block rather than across experimental blocks: the crucial result is the advantage for unconnected over connected targets, and this result was observed within one experimental block. Such an effect has never been observed in trained subjects, whatever the blocks order. Nor was it observed in controls in the present experiment. It can thus be reasonably estimated as being independent of this methodological limitation, especially as it replicates a similar result observed with a different paradigm (Giersch et al., 2011).

It might also be questioned if the loss of perceptual organization observed in patients is specifically related to the need to re-group unconnected items, or whether it reflects a more general weakness in grouping by connectors.

The amplitude of the reversed advantage for unconnected targets in patients shows that the experiment is sensitive enough to reveal a general weakness in automatic grouping. Weakened automatic grouping should have reduced the performance advantage provided by the connecters across all experimental blocks. However, when patients did not prioritize unconnected pairs, they displayed a preserved benefit for connected over unconnected targets. In the present results, this advantage was rather larger in patients than in controls when the prioritization concerned connected pairs. In addition the advantage for connected pairs was preserved in case of across-hemifield presentation. In sum, weakened grouping by connectors appears to result from prior attention focus on unconnected pairs rather than a genuine impairment in automatic grouping, consistent with previous results (Giersch and Rhein, 2008; Giersch et al., 2011; van Assche and Giersch, 2011).

Contrary to controls, however, patients showed significantly different effects in case of within- vs. across-hemifield presentation. Such an effect of hemifield presentation has not been observed in healthy subjects, neither in the present study, nor in our previous study (van Assche et al., 2012). Even when probability effects led to clear performance improvements for unconnected figures in healthy subjects (van Assche et al., 2012), this improvement was similar whatever the position of the targets (within- or across-hemifields). Importantly this coexisted with a high cost of across-hemifield presentation for unconnected figures, showing that the paradigm was sensitive to the cost of across-hemifield presentation. This pattern of results is in marked contrast with the results of patients in the present study. These results suggest that even if patients “re-group” information, they do not do it in the same way as healthy subjects do. The fact that patients “re-group” efficiently only in case of within-hemifield, and not across-hemifield presentation, might be explained by an involvement of the connectivity between hemispheres. This difference between patients and controls can hardly be explained by a difference in the cost of interhemispheric transfer. There was no evidence of a higher cost of across-hemifield presentation in patients than in controls, and the main difference between the two groups occurred in case of intra-hemifield presentation. The results rather suggest that controls mobilize lateralized mechanisms when prioritizing unconnected figures (Kosslyn, 1987; van der Ham et al., 2011; Stevens et al., 2012), whereas patients use mechanisms requiring an exchange of information between hemispheres, i.e., possibly earlier and more automatic mechanisms. This interpretation requires confirmation. However, whatever the precise explanations for the effects in patients, they suggest that the patients do not re-group items in the same way as controls. In other perception studies (Giersch and Rhein, 2008; van Assche and Giersch, 2011), patients did not show evidence of “re-grouping,” but the results again suggested difficulties with re-grouping. All in all these results confirm that re-grouping requires specific mechanisms and, most importantly, that preserving automatic grouping when re-grouping unconnected figures is not straightforward.

### Integrating grouping and “re-grouping”: Our proposal

We argue that the results observed in patients with schizophrenia shed light on the difficulties encountered when exploring the visual environment in a flexible way while maintaining a sensation of stability of the outer world. The results in patients emphasize the difficulty arising when relating unconnected items in the environment. Our results show that being able to re-group items is not enough to explore visual information in an optimal way, i.e., without loosing access to automatically grouped figures. First, the way re-grouping is performed matters. It is important that grouping and re-grouping are based on distinct mechanisms. Our previous study had already suggested that the two types of grouping are not only based on different pathways, but also lead to different outputs (van Assche et al., 2012). This then raises other questions, however: what is the output of re-grouping, and how prioritizing re-grouping coexists with easy access to automatically grouped items. If the outputs of grouping and re-grouping are distinct and accessed in parallel, there should be some innate priority given to automatic grouping. This way access to items issued from automatic grouping would be preserved even when the subject prioritizes re-grouped items. This might not be enough, however. Additional ties might be necessary between the two types of groupings. Even if “re-grouping” mainly involves high-level cognitive mechanisms, these mechanisms allow us to play with information that is continuously processed by our visual system. For example, when we mentally select two tangerines from different piles and thus mentally separate them from their piles, we still see these tangerines as belonging to their piles. When having compared the tangerines and made a choice, we need to know from which piles they are issued to take the chosen tangerines. It might thus be proposed that the “re-grouping” of items integrates the links these items have with connected objects. This would mean strong ties between re-grouped items and automatically grouped ones. The literature suggests the possibility of a more integrated representation. As emphasized above, several studies suggest the existence of specialized areas sub-tending the coding of spatial and conceptual relationships between objects (Kosslyn, 1987; van der Ham et al., 2011; Stevens et al., 2012). In line with this specialization, a possibility would be the building of a complex representation integrating the usual links issued from automatic grouping with the links created when mentally “re-grouping” objects. This would allow access to both connected and unconnected items.

Such a representation goes beyond the hierarchical representations involving local and global information. As we have seen, there are pathways specialized in the processing of local and global information and both types of information are first processed through bottom-up, automatic mechanisms. In fact they can be considered as part of the automatic grouping processes. This is not the case for “re-grouping,” however. As already emphasized, “re-grouped” pairs correspond neither to local elements nor to global information. In contrast with local/global processing, the creation of a link between two shapes that belong to different groups thus primarily originates from attention mechanisms, and creating such links is costly. It might be considered as an extension of the coding of independent elements as described by Humphreys (1998). In this work, it was proposed that “visual elements can be selected together provided that the elements activate a single, stored object representation.” In our case, a representation of “re-grouped” items does not pre-exist to the task. We propose however that subjects build this representation as a result of the task at hand. Although this idea clearly requires confirmation, it is supported by several observations. First, results in patients with schizophrenia suggest that patients avoid “re-grouping” when possible (Giersch and Rhein, 2008; van Assche and Giersch, 2011), possibly due to the effort it entails. Second, when patients cannot avoid “re-grouping,” they probably do it differently from controls, and experience a conflict between automatic grouping and “re-grouping,” which suggests that the integration of both types of groupings represents an additional cost. All in all, this might suggest the existence of specific mechanisms in order to integrate the link between “re-grouped” items in the representation of the visual scene. Some kind of relationship must be established between the two types of pairs in order to allow for both a selective focalization on one type of pair and an easy access to both. It is not only the new link between “re-grouped” items that would be coded, but also how they are related with other pairs. An item from a given set that is re-grouped with an other item from another set would be tagged as “re-grouped” but also as being part of a set of objects. Thus, when prioritizing “re-grouped” items, one would select a pair of figures tagged as being part of different groups. This representation is necessarily complex, since it includes the coding of conflicting links between objects. It is probably costly to build such a representation, but once built it enables a flexible exploration of the outer world while maintaining its stability. This is precisely what seems to be impaired in patients with schizophrenia.

### Limits and perspectives

Our proposal regarding how “re-grouping” is tied in with automatic grouping clearly requires confirmation. It remains also to be understood how a complex representation integrating outputs of “re-grouping” and automatic grouping fits in with hierarchical representations issued from local and global information. It might be possible that links issued from “re-grouping” represent an additional level of complexity that would be integrated with hierarchical representations through learning, thus leading to the building of the complex representations we propose here. This question is important in order to understand how “re-grouping” impacts on the exploration of visual scenes. It might be possible to study this question by checking to which amount the impairments described here in patients with schizophrenia are at the origin of their reduced span of exploration when spontaneously looking at visual objects or scenes (Gaebel et al., 1987; Kojima et al., 1990; Gordon et al., 1992; Phillips and David, 1997; Loughland et al., 2002; Obayashi et al., 2003; Minassian et al., 2005; Delerue et al., 2010; Delerue and Boucart, 2012). As a rule, patients’ span of exploration is reduced in space and the duration of their fixations is longer (Gaebel et al., 1987; Kojima et al., 1990; Gordon et al., 1992; Phillips and David, 1997; Loughland et al., 2002; Obayashi et al., 2003; Minassian et al., 2005). More often than not, they focus on non-significant details, and explore one part of a stimulus while missing important parts of the faces or objects (Obayashi et al., 2003; Minassian et al., 2005). In order to explore the environment in a coherent way, one needs to be able to go from one object to another without losing the visual scene from sight (Bullier, 2001; Fenske et al., 2006; Huang and Grossberg, 2010; Peyrin et al., 2010). Patients precisely appear to be impaired at relating unconnected items without losing basic links from sight. This might also account for their own complaints (Chapman, 1966): “Everything I see is split up. It’s like a photograph that’s torn in bits and put together again. If somebody moves or speaks, everything I see disappears quickly and I have to put it together again.”

It will be especially of interest to understand how complex representations are used to guide visual exploration. It is known that eye movements are not only automatic responses to retinal inputs but are regulated by a process of target selection involving a variety of complex processes, including attention, perception, memory, and expectation (Henderson and Hollingworth, 2003; Hopp and Fuchs, 2004; Krauzlis, 2005; Iwamoto and Kaku, 2010; Pélisson et al., 2010). It remains to be seen to which amount visual re-grouping is part of these mechanisms and affects endogenously driven visual exploration.

## Conclusion

Results in patients with schizophrenia and in healthy volunteers suggest that it is possible to mentally re-group items from different sets of objects. This re-grouping conflicts with usual grouping issued from automatic grouping, and requires a cognitive processing that differs from usual grouping and from local vs. global processing. The conflict between the two types of groupings is evidenced in patients with schizophrenia. Trained healthy volunteers, however, appear to process re-grouped objects while preserving easy access to automatically grouped objects. We propose that easy access to both types of grouping is enabled by the building of a complex representation integrating the relationships between “re-grouped” and grouped objects.

## Conflict of Interest Statement

The authors declare that the research was conducted in the absence of any commercial or financial relationships that could be construed as a potential conflict of interest.
